# Repetitive Transcranial Magnetic Stimulation (rTMS) for Smoking Cessation: A Systematic Review of Randomized Controlled Trials

**DOI:** 10.1192/j.eurpsy.2026.10730

**Published:** 2026-07-31

**Authors:** S. Jaka, T. Barias, S. Prasad, S. Kaur, O. ElShahawy

**Affiliations:** 1 Psychiatry and Behavioral Health Sciences, Nassau Univeristy Medical center, East Meadow; 2 Psychiatry, Bronxcare Health system, Bronx; 3 Section on Tobacco, Alcohol, and Drug Use, Department of Population Health, School of Medicine, New York University, New york, United States

## Abstract

**Introduction:**

Traditional smoking cessation methods—including pharmacotherapies and behavioral therapies—often fall short in achieving long-term abstinence. Pharmacologic interventions may not outperform placebo in real-world settings, and the safety and effectiveness of alternatives like e-cigarettes remain uncertain. Given the neurobiological underpinnings of nicotine addiction, repetitive transcranial magnetic stimulation (rTMS) has emerged as a promising noninvasive intervention targeting brain regions involved in craving, reward, and executive control.

**Objectives:**

To systematically evaluate the efficacy and safety of rTMS, including newer protocols such as intermittent theta burst stimulation (iTBS) and deep TMS (dTMS), for smoking cessation

**Methods:**

We searched MEDLINE, Embase, PsycINFO, and CINAHL through March 2025 for English-language RCTs involving adult smokers treated with rTMS versus sham. Outcomes of interest included smoking abstinence rates, cigarette consumption, craving, and adverse events. Screening and data extraction were conducted by three independent reviewers using Rayyan, with conflicts resolved by consensus.

**Results:**

Seventeen studies involving 956 participants met inclusion criteria. Among them, 491 received active rTMS, and 448 received sham stimulation (data unavailable for 17 participants). The majority targeted the left dorsolateral prefrontal cortex (L-DLPFC), with protocols varying in frequency (1–20 Hz), intensity (80–120% rMT), and number of sessions (1 to 21). One study used iTBS, and two employed deep TMS targeting bilateral DLPFC and insula.

Cigarette consumption and craving significantly decreased in most active treatment arms compared to sham. Biochemically verified abstinence was significantly higher in select studies, particularly those using deep TMS. However, treatment effects often declined by 3- to 6-month follow-up. Across all trials, rTMS was well-tolerated, with no serious adverse events reported.

**Conclusions:**

rTMS is a safe and potentially effective neuromodulatory intervention for reducing cigarette use, craving, and nicotine dependence. While short-term benefits are promising, long-term efficacy remains uncertain, and heterogeneity in protocols complicates direct comparisons. Deep TMS and theta burst protocols warrant further exploration in large-scale trials, particularly in diverse populations and those with comorbid psychiatric conditions.

**Disclosure of Interest:**

None Declared

